# Crystal structure of tetra­aqua­(5,5′-dimethyl-2,2′-bipyridyl-κ^2^ *N*,*N*′)iron(II) sulfate

**DOI:** 10.1107/S1600536814024982

**Published:** 2014-11-21

**Authors:** Yamine Belamri, Fatima Setifi, Bojana M. Francuski, Sladjana B. Novaković, Setifi Zouaoui

**Affiliations:** a Laboratoire de Chimie, Ingénierie Moléculaire et Nanostructures (LCIMN), Université Ferhat Abbas Sétif 1, Sétif 19000, Algeria; b Vinča Institute of Nuclear Sciences, Laboratory of Theoretical Physics and Condensed Matter Physics, PO Box 522, University of Belgrade, 11001 Belgrade, Serbia; c Département de Technologie, Faculté de Technologie, Université 20 Août 1955-Skikda, BP 26, Route d’El-Hadaiek, Skikda 21000, Algeria; d Unité de Recherche de Chimie de l’Environnement et Moléculaire Structurale (CHEMS), Université Constantine 1, Constantine 25000, Algeria

**Keywords:** crystal structure, 5,5′-dimethyl-2,2′-dipyrid­yl, tetra­aqua­iron(II) complex, sulfate, bi­pyridine ligand, hydrogen bonding, π–π inter­actions

## Abstract

In the title compound, [Fe(C_12_H_12_N_2_)(H_2_O)_4_]SO_4_, the central Fe^II^ ion is coordinated by two N atoms from the 5,5′-dimethyl-2,2′-bi­pyridine ligand and four water O atoms in a distorted octa­hedral geometry. The Fe—O coordination bond lengths vary from 2.080 (3) to 2.110 (3) Å, while the two Fe—N coordination bonds have practically identical lengths [2.175 (3) and 2.177 (3) Å]. The chelating N—Fe—N angle of 75.6 (1)° shows the largest deviation from an ideal octa­hedral geometry; the other coordination angles deviate from ideal values by 0.1 (1) to 9.1 (1)°. O—H⋯O hydrogen bonding between the four aqua ligands of the cationic complex and four O-atom acceptors of the anion leads to the formation of layers parallel to the *ab* plane. Neighbouring layers further inter­act by means of C—H⋯O and π–π inter­actions involving the laterally positioned bi­pyridine rings. The perpen­dicular distance between π–π inter­acting rings is 3.365 (2) Å, with a centroid–centroid distance of 3.702 (3) Å.

## Abstract 2

In the crystal structure of the title compound, [Fe(dmbpy)(H_2_O)_4_][SO4], the charged components form an extensive hydrogen-bonding network. Eight O—H⋯O hydrogen bonds [*d*(O⋯H) < 2.00 Å], form a two-dimensional network parallel to the *ab* plane.

## Chemical context   

Coordination compounds containing polynitrile anions as ligands are of current inter­est for their magnetic properties and their rich architectures and topologies (Setifi *et al.*, 2003 ▶; Gaamoune *et al.*, 2010 ▶; Váhovská & Potočňák, 2012 ▶; Setifi, Setifi *et al.*, 2013 ▶; Setifi, Domasevitsch *et al.*, 2013 ▶; Potočňák *et al.*, 2014 ▶). Given the crucial role of these anionic ligands, we are inter­ested in using them in combination with other chelating or bridging neutral co-ligands to explore their structural and electronic characteristics in the large field of mol­ecular materials exhibiting the spin crossover (SCO) phenomenon. In an attempt to prepare such a complex, we obtained the title compound, [Fe(dmbpy)(H_2_O)_4_]SO_4_, (I), where dmbpy is 5,5′-dimethyl-2,2′-bipyridyl. 
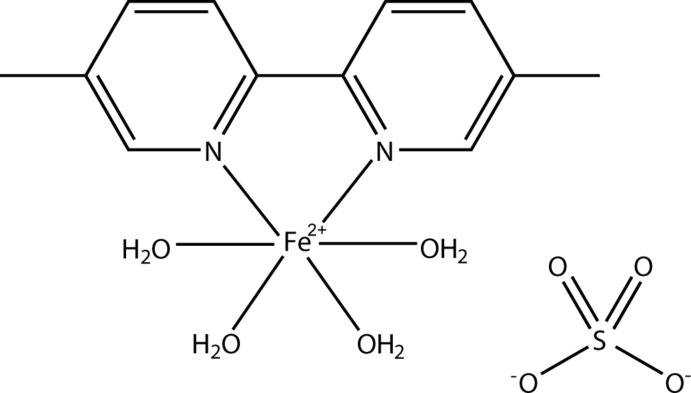


 The crystal structures of several complexes with general formula [*M*(bpy)(H_2_O)_4_]^2+^ comprising bipyridine derivatives as ligands have been reported previously (Boonlue *et al.*, 2012 ▶; Harvey *et al.*, 1999 ▶; Kwak *et al.*, 2007 ▶; Suarez *et al.*, 2013 ▶; Xiao *et al.*, 2003 ▶; Yang, 2009 ▶; Yu *et al.*, 2007 ▶; Zhang *et al.*, 2008 ▶; Zhao & Bai, 2009 ▶). This is the first complex of this type with Fe^II^ as the central ion.

## Structural commentary   

**Figure 1:**
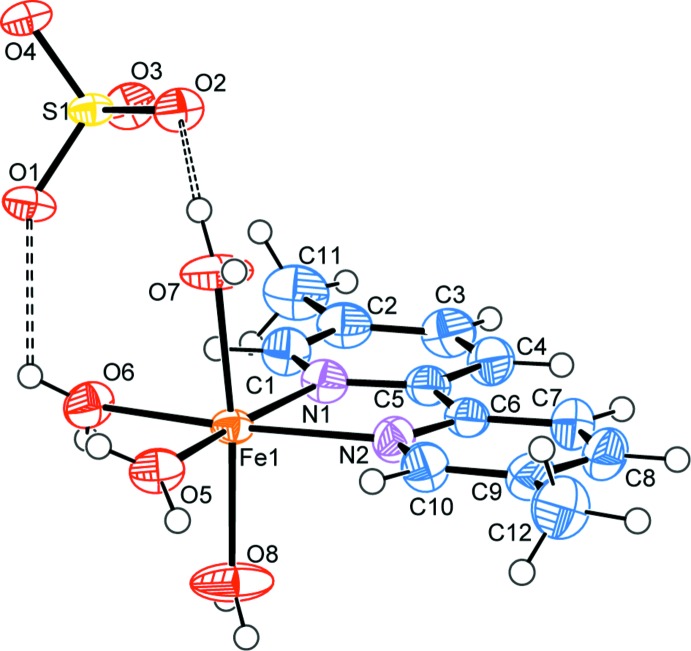
The mol­ecular structure of (I), with atom labels and 50% probability displacement ellipsoids for non-H atoms. Hydrogen bonds are indicated by dashed lines.

A mol­ecular view of complex (I), together with the atom-numbering scheme is given in Fig. 1 ▶. The crystal structure of (I) consists of the cationic complex [Fe(dmbpy)(H_2_O)_4_]^2+^ and a free [SO_4_]^2−^ counter-ion. The Fe^II^ atom is in a distorted octa­hedral coordination environment and the equatorial plane of the octa­hedron is formed by a pair of nitro­gen donors from the 5,5′-dimethyl-2,2′-bipyridyl ligand and two mol­ecules of water, while the axial sites are occupied by two other water mol­ecules. The equatorial donor atoms are nearly coplanar (r.m.s. deviation = 0.0062 Å), while the deviation of the Fe atom from the least-squares plane is somewhat larger [0.021 (2) Å]. The bi­pyridine chelating angle N1—Fe—N2 of 75.6 (1)° shows the most significant deviation from an ideal octa­hedral geometry. The other angular distortions from an ideal octa­hedral geometry are in the range 0.1 (1) to 9.1 (1)°. The S—O bond lengths [1.466 (3)–1.480 (3) Å] and O—S—O angles [108.8 (2)–109.9 (2)°] indicate a nearly ideal tetra­hedral geometry for the anion.

## Supra­molecular features   

**Figure 2:**
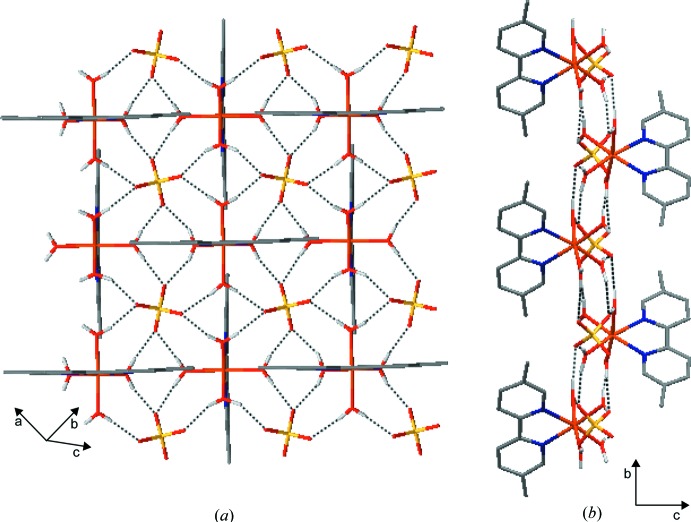
(*a*) O—H⋯O inter­actions (dashed lines) connect the cations and anions into layers parallel to the *ab* plane. (*b*) View of a single layer down the *a* axis.

**Figure 3:**
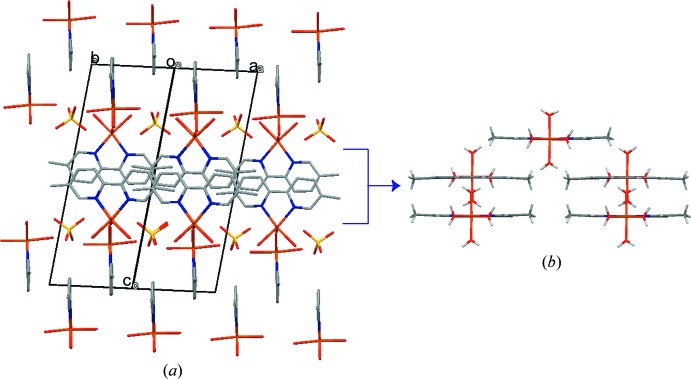
(*a*) The bipyridine rings from neighbouring layers inter­act *via* C—H⋯O and π–π inter­actions. (*b*) Orthogonal projection of the central fragment.

Within the crystal packing, the charged components are connected by an extensive hydrogen-bonding network (Table 1 ▶). Each of the [Fe(dmbpy)(H_2_O)_4_]^2+^ cations engages all four coordinating water mol­ecules in hydrogen bonding to four [SO_4_]^2−^ anions (Fig. 2 ▶ *a*). The anions surrounding the cationic unit are positioned at similar Fe⋯S distance of 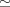
4.9 Å. On the other hand, each of the [SO_4_]^2−^ anions appears surrounded with four cationic units, where its four O atoms engage as acceptors in bifurcated O—H⋯O hydrogen bonds towards neighbouring cations (Fig. 2 ▶ *a*). Such a mutual arrangement leads to the formation of a two-dimensional hydrogen-bonded network parallel to the *ab* plane (Fig. 2 ▶ *b*). Laterally arranged aromatic rings of the 5,5′-dimethyl-2,2′-bi­pyridine ligand in neighbouring layers inter­act by means of weak C—H⋯O and π–π inter­actions, forming the three-dimensional crystal packing (Table 1 ▶ and Fig. 3 ▶). The centroid–centroid distance for the latter inter­action is 3.702 (3) Å.

## Synthesis and crystallization   

The title compound, (I), was synthesized hydro­thermally from a mixture of iron(II) sulfate hepta­hydrate (28 mg, 0.1 mmol), 5,5′-dimethyl-2,2′-bipyridyl (18 mg, 0.1 mmol) and potassium tri­cyano­methanide KC(CN)_3_ (26 mg, 0.2 mmol) in water–ethanol (4:1 *v*/*v*, 20 ml). The mixture was transferred to a Teflon-lined autoclave and heated at 410 K for 3 d. The autoclave was then allowed to cool to ambient temperature. Red crystals of (I) were collected by filtration, washed with water and dried in air (yield 35%).

## Refinement details   

Crystal data, data collection and structure refinement details are summarized in Table 2 ▶. H atoms bonded to C atoms were placed at geometrically calculated positions and refined using a riding model. C—H distances were fixed at 0.93 and 0.96 Å from aromatic and methyl C atoms, respectively. The *U*_iso_(H) values were equal to 1.2 and 1.5 times *U*_eq_ of the corresponding C(*sp* ^2^) and C(*sp* ^3^) atoms. The H atoms of the four water mol­ecules were initially located in a difference Fourier map. During the refinement, these H atoms were allowed to ride on their parent O atoms and also to rotate about the corresponding Fe—O bonds. The *U*_iso_(H) values were set equal to 1.2 times *U*_eq_ of the parent O atom. The reflections (100) and (002) were excluded from the refinement because they were nearly completely obscured by the beamstop.

## Supplementary Material

Crystal structure: contains datablock(s) global, I. DOI: 10.1107/S1600536814024982/vn2087sup1.cif

Structure factors: contains datablock(s) I. DOI: 10.1107/S1600536814024982/vn2087Isup2.hkl

CCDC reference: 1034106

Additional supporting information:  crystallographic information; 3D view; checkCIF report

## Figures and Tables

**Table 1:** Hydrogen-bond geometry (, )

*D*H*A*	*D*H	H*A*	*D* *A*	*D*H*A*
O5H1*O*5O4^i^	0.83	1.92	2.734(4)	165
O5H2*O*5O2^ii^	0.96	1.94	2.794(4)	147
O6H1*O*6O1	0.94	1.90	2.820(4)	167
O6H2*O*6O3^iii^	0.83	1.95	2.765(4)	165
O7H1*O*7O4^ii^	0.83	1.89	2.722(4)	175
O7H2*O*7O2	0.82	1.89	2.697(4)	167
O8H1*O*8O1^iii^	0.77	1.95	2.719(4)	175
O8H2*O*8O3^i^	0.89	1.91	2.792(5)	174
C4H4O4^iv^	0.93	2.54	3.232(5)	132

Symmetry codes: (i) 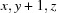; (ii) 
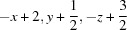
; (iii) 
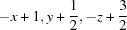
; (iv) 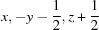.

**Table 2:** Experimental details

Crystal data	
Chemical formula	[Fe(C_12_H_12_N_2_)(H_2_O)_4_]SO_4_
*M*_r_	408.21
Crystal system, space group	Monoclinic, *P*2_1_/*c*
Temperature (K)	293
*a*, *b*, *c* ()	9.5790(7), 9.6190(9), 18.5500(12)
()	101.527(5)
*V* (^3^)	1674.7(2)
*Z*	4
Radiation type	Mo *K*
(mm^1^)	1.07
Crystal size (mm)	0.28 0.14 0.09

Data collection	
Diffractometer	Bruker APEXII CCD
Absorption correction	Multi-scan (*SADABS*; Bruker, 2009 ▶)
*T*_min_, *T*_max_	0.792, 0.881
No. of measured, independent and observed [*I* > 2(*I*)] reflections	14477, 4868, 3305
*R*_int_	0.117
(sin /)_max_ (^1^)	0.706

Refinement	
*R*[*F* ^2^ > 2(*F* ^2^)], *wR*(*F* ^2^), *S*	0.065, 0.196, 1.08
No. of reflections	4867
No. of parameters	223
H-atom treatment	H-atom parameters constrained
_max_, _min_ (e ^3^)	0.84, 1.33

Computer programs: *APEX2* and *SAINT* (Bruker, 2009 ▶), *SHELXS97* (Sheldrick, 2008 ▶), *SHELXL2014* (Sheldrick, 2008 ▶), *ORTEP-3 for Windows* (Farrugia, 2012 ▶) and *Mercury* (Macrae *et al.*, 2006 ▶).

## supplementary crystallographic information

### Crystal data

**Table:**

[Fe(C_12_H_12_N_2_)(H_2_O)_4_]SO_4_	*F*(000) = 848
*M**_r_* = 408.21	*D*_x_ = 1.619 Mg m^−^^3^
Monoclinic, *P*2_1_/*c*	Mo *K*α radiation, λ = 0.71073 Å
Hall symbol: -P 2ybc	Cell parameters from 4857 reflections
*a* = 9.5790 (7) Å	θ = 2.2–29.9°
*b* = 9.6190 (9) Å	µ = 1.07 mm^−^^1^
*c* = 18.5500 (12) Å	*T* = 293 K
β = 101.527 (5)°	Block, red
*V* = 1674.7 (2) Å^3^	0.28 × 0.14 × 0.09 mm
*Z* = 4	

### Data collection

**Table:**

Bruker APEXII CCD diffractometer	4868 independent reflections
Radiation source: fine-focus sealed tube	3305 reflections with *I* > 2σ(*I*)
Graphite monochromator	*R*_int_ = 0.117
ω–2θ scans	θ_max_ = 30.1°, θ_min_ = 2.2°
Absorption correction: multi-scan (*SADABS*; Bruker, 2009)	*h* = −13→13
*T*_min_ = 0.792, *T*_max_ = 0.881	*k* = −13→13
14477 measured reflections	*l* = −26→26

### Refinement

**Table:**

Refinement on *F*^2^	223 parameters
Least-squares matrix: full	H-atom parameters constrained
*R*[*F*^2^ > 2σ(*F*^2^)] = 0.065	*w* = 1/[σ^2^(*F*_o_^2^) + (0.0821*P*)^2^ + 2.0315*P*] where *P* = (*F*_o_^2^ + 2*F*_c_^2^)/3
*wR*(*F*^2^) = 0.196	(Δ/σ)_max_ = 0.002
*S* = 1.08	Δρ_max_ = 0.84 e Å^−^^3^
4867 reflections	Δρ_min_ = −1.33 e Å^−^^3^

### Special details

**Table:**

Geometry. All esds (except the esd in the dihedral angle between two l.s. planes) are estimated using the full covariance matrix. The cell esds are taken into account individually in the estimation of esds in distances, angles and torsion angles; correlations between esds in cell parameters are only used when they are defined by crystal symmetry. An approximate (isotropic) treatment of cell esds is used for estimating esds involving l.s. planes.

### Fractional atomic coordinates and isotropic or equivalent isotropic displacement parameters (Å^2^)

**Table:**

	*x*	*y*	*z*	*U*_iso_*/*U*_eq_	
Fe1	0.77171 (5)	0.13308 (6)	0.80535 (3)	0.02534 (17)	
S1	0.75114 (9)	−0.36313 (10)	0.75262 (5)	0.0251 (2)	
O3	0.6693 (3)	−0.4269 (3)	0.80282 (17)	0.0376 (7)	
O1	0.6620 (3)	−0.2600 (3)	0.70522 (17)	0.0364 (7)	
O2	0.8783 (3)	−0.2931 (3)	0.79546 (16)	0.0338 (6)	
O4	0.7980 (3)	−0.4722 (3)	0.70641 (16)	0.0347 (6)	
O5	0.8418 (3)	0.2477 (3)	0.72229 (16)	0.0361 (7)	
H1O5	0.8129	0.3296	0.7171	0.043*	
H2O5	0.9194	0.2065	0.7043	0.043*	
O6	0.6213 (3)	0.0261 (3)	0.72705 (17)	0.0399 (7)	
H1O6	0.6389	−0.0652	0.7130	0.048*	
H2O6	0.5378	0.0556	0.7195	0.048*	
O7	0.9191 (3)	−0.0166 (3)	0.7871 (2)	0.0489 (9)	
H1O7	1.0046	−0.0016	0.7863	0.059*	
H2O7	0.8929	−0.0981	0.7879	0.059*	
O8	0.6224 (3)	0.2876 (4)	0.8109 (2)	0.0619 (11)	
H1O8	0.5422	0.2701	0.8053	0.074*	
H2O8	0.6333	0.3793	0.8107	0.074*	
N1	0.7224 (4)	0.0244 (4)	0.90015 (19)	0.0340 (8)	
N2	0.9173 (4)	0.2218 (4)	0.89908 (19)	0.0337 (8)	
C1	0.6251 (5)	−0.0763 (5)	0.8969 (3)	0.0390 (10)	
H1	0.5747	−0.1028	0.8508	0.047*	
C2	0.5945 (5)	−0.1437 (5)	0.9581 (3)	0.0424 (10)	
C3	0.6691 (6)	−0.0975 (5)	1.0262 (3)	0.0452 (11)	
H3	0.6497	−0.1362	1.0691	0.054*	
C4	0.7709 (5)	0.0043 (5)	1.0307 (2)	0.0412 (10)	
H4	0.8219	0.0327	1.0763	0.049*	
C5	0.7973 (4)	0.0650 (5)	0.9666 (2)	0.0341 (9)	
C6	0.9054 (4)	0.1741 (4)	0.9658 (2)	0.0316 (8)	
C7	0.9931 (5)	0.2257 (5)	1.0288 (2)	0.0440 (11)	
H7	0.9845	0.1926	1.0748	0.053*	
C8	1.0931 (5)	0.3265 (5)	1.0230 (3)	0.0436 (11)	
H8	1.1512	0.3617	1.0653	0.052*	
C9	1.1071 (5)	0.3750 (5)	0.9549 (3)	0.0411 (10)	
C10	1.0156 (5)	0.3184 (5)	0.8947 (2)	0.0381 (9)	
H10	1.0231	0.3496	0.8482	0.046*	
C11	0.4852 (6)	−0.2566 (6)	0.9492 (3)	0.0594 (14)	
H11A	0.3987	−0.2243	0.9184	0.089*	
H11B	0.4670	−0.2817	0.9966	0.089*	
H11C	0.5198	−0.3363	0.9270	0.089*	
C12	1.2165 (5)	0.4814 (6)	0.9444 (3)	0.0553 (13)	
H12A	1.2919	0.4366	0.9261	0.083*	
H12B	1.2548	0.5249	0.9907	0.083*	
H12C	1.1726	0.5504	0.9099	0.083*	

### Atomic displacement parameters (Å^2^)

**Table:**

	*U*^11^	*U*^22^	*U*^33^	*U*^12^	*U*^13^	*U*^23^
Fe1	0.0228 (3)	0.0200 (3)	0.0332 (3)	0.0001 (2)	0.00542 (19)	0.0011 (2)
S1	0.0219 (4)	0.0171 (4)	0.0374 (5)	0.0005 (3)	0.0084 (3)	0.0000 (4)
O3	0.0387 (16)	0.0309 (16)	0.0461 (17)	−0.0077 (13)	0.0152 (13)	0.0018 (13)
O1	0.0323 (15)	0.0262 (15)	0.0500 (17)	0.0075 (12)	0.0067 (12)	0.0064 (13)
O2	0.0223 (12)	0.0289 (16)	0.0499 (17)	−0.0067 (11)	0.0065 (11)	−0.0046 (13)
O4	0.0371 (15)	0.0236 (14)	0.0455 (16)	0.0052 (12)	0.0135 (12)	−0.0051 (12)
O5	0.0361 (15)	0.0212 (14)	0.0536 (18)	−0.0039 (12)	0.0151 (13)	0.0065 (13)
O6	0.0266 (14)	0.0299 (16)	0.0601 (19)	−0.0038 (12)	0.0013 (13)	−0.0091 (15)
O7	0.0286 (15)	0.0234 (15)	0.098 (3)	0.0025 (12)	0.0207 (17)	0.0004 (17)
O8	0.0270 (16)	0.0275 (17)	0.134 (4)	0.0013 (13)	0.022 (2)	0.000 (2)
N1	0.0345 (17)	0.0336 (19)	0.0351 (18)	0.0003 (15)	0.0102 (14)	0.0042 (15)
N2	0.0352 (18)	0.0307 (18)	0.0336 (17)	−0.0043 (14)	0.0033 (14)	−0.0016 (14)
C1	0.039 (2)	0.036 (2)	0.042 (2)	−0.0060 (19)	0.0111 (18)	0.0020 (19)
C2	0.045 (2)	0.035 (2)	0.051 (3)	0.001 (2)	0.017 (2)	0.009 (2)
C3	0.059 (3)	0.042 (3)	0.040 (2)	0.002 (2)	0.024 (2)	0.012 (2)
C4	0.050 (3)	0.043 (3)	0.033 (2)	0.003 (2)	0.0133 (19)	0.0032 (19)
C5	0.037 (2)	0.032 (2)	0.034 (2)	0.0064 (17)	0.0093 (16)	0.0015 (17)
C6	0.0318 (19)	0.030 (2)	0.034 (2)	0.0048 (16)	0.0078 (16)	0.0014 (16)
C7	0.049 (3)	0.047 (3)	0.034 (2)	0.002 (2)	0.0034 (19)	−0.003 (2)
C8	0.044 (2)	0.040 (3)	0.044 (3)	−0.005 (2)	0.0012 (19)	−0.012 (2)
C9	0.040 (2)	0.035 (2)	0.047 (3)	−0.0016 (19)	0.0050 (18)	−0.005 (2)
C10	0.040 (2)	0.036 (2)	0.038 (2)	−0.0048 (19)	0.0059 (17)	0.0020 (19)
C11	0.061 (3)	0.050 (3)	0.073 (4)	−0.006 (3)	0.028 (3)	0.014 (3)
C12	0.042 (3)	0.051 (3)	0.069 (3)	−0.017 (2)	0.003 (2)	−0.007 (3)

### Geometric parameters (Å, º)

**Table:**

Fe1—O8	2.080 (3)	C1—H1	0.9300
Fe1—O7	2.091 (3)	C2—C3	1.394 (7)
Fe1—O6	2.099 (3)	C2—C11	1.494 (7)
Fe1—O5	2.110 (3)	C3—C4	1.373 (7)
Fe1—N2	2.175 (3)	C3—H3	0.9300
Fe1—N1	2.177 (3)	C4—C5	1.392 (6)
S1—O3	1.466 (3)	C4—H4	0.9300
S1—O2	1.477 (3)	C5—C6	1.477 (6)
S1—O1	1.479 (3)	C6—C7	1.388 (6)
S1—O4	1.480 (3)	C7—C8	1.382 (7)
O5—H1O5	0.8346	C7—H7	0.9300
O5—H2O5	0.9588	C8—C9	1.379 (7)
O6—H1O6	0.9409	C8—H8	0.9300
O6—H2O6	0.8339	C9—C10	1.385 (6)
O7—H1O7	0.8346	C9—C12	1.504 (7)
O7—H2O7	0.8248	C10—H10	0.9300
O8—H1O8	0.7727	C11—H11A	0.9600
O8—H2O8	0.8889	C11—H11B	0.9600
N1—C1	1.337 (6)	C11—H11C	0.9600
N1—C5	1.354 (5)	C12—H12A	0.9600
N2—C10	1.336 (5)	C12—H12B	0.9600
N2—C6	1.345 (5)	C12—H12C	0.9600
C1—C2	1.389 (6)		
			
O8—Fe1—O7	173.50 (16)	C2—C1—H1	117.9
O8—Fe1—O6	90.06 (14)	C1—C2—C3	116.0 (4)
O7—Fe1—O6	86.71 (13)	C1—C2—C11	120.5 (5)
O8—Fe1—O5	89.18 (14)	C3—C2—C11	123.6 (4)
O7—Fe1—O5	85.26 (13)	C4—C3—C2	120.7 (4)
O6—Fe1—O5	91.46 (12)	C4—C3—H3	119.6
O8—Fe1—N2	90.98 (15)	C2—C3—H3	119.6
O7—Fe1—N2	93.10 (14)	C3—C4—C5	119.7 (4)
O6—Fe1—N2	170.92 (13)	C3—C4—H4	120.1
O5—Fe1—N2	97.58 (13)	C5—C4—H4	120.1
O8—Fe1—N1	92.32 (15)	N1—C5—C4	120.2 (4)
O7—Fe1—N1	93.60 (14)	N1—C5—C6	116.1 (4)
O6—Fe1—N1	95.39 (13)	C4—C5—C6	123.7 (4)
O5—Fe1—N1	172.99 (13)	N2—C6—C7	120.3 (4)
N2—Fe1—N1	75.55 (14)	N2—C6—C5	116.1 (4)
O3—S1—O2	109.72 (18)	C7—C6—C5	123.6 (4)
O3—S1—O1	109.88 (18)	C8—C7—C6	119.8 (4)
O2—S1—O1	109.25 (18)	C8—C7—H7	120.1
O3—S1—O4	109.51 (18)	C6—C7—H7	120.1
O2—S1—O4	108.76 (17)	C9—C8—C7	120.3 (4)
O1—S1—O4	109.70 (18)	C9—C8—H8	119.8
Fe1—O5—H1O5	115.4	C7—C8—H8	119.8
Fe1—O5—H2O5	114.8	C8—C9—C10	116.3 (4)
H1O5—O5—H2O5	127.9	C8—C9—C12	123.1 (4)
Fe1—O6—H1O6	120.9	C10—C9—C12	120.6 (4)
Fe1—O6—H2O6	116.9	N2—C10—C9	124.4 (4)
H1O6—O6—H2O6	119.3	N2—C10—H10	117.8
Fe1—O7—H1O7	125.5	C9—C10—H10	117.8
Fe1—O7—H2O7	115.7	C2—C11—H11A	109.5
H1O7—O7—H2O7	118.0	C2—C11—H11B	109.5
Fe1—O8—H1O8	120.9	H11A—C11—H11B	109.5
Fe1—O8—H2O8	128.8	C2—C11—H11C	109.5
H1O8—O8—H2O8	109.3	H11A—C11—H11C	109.5
C1—N1—C5	119.2 (4)	H11B—C11—H11C	109.5
C1—N1—Fe1	125.0 (3)	C9—C12—H12A	109.5
C5—N1—Fe1	115.8 (3)	C9—C12—H12B	109.5
C10—N2—C6	118.9 (4)	H12A—C12—H12B	109.5
C10—N2—Fe1	124.9 (3)	C9—C12—H12C	109.5
C6—N2—Fe1	116.3 (3)	H12A—C12—H12C	109.5
N1—C1—C2	124.1 (4)	H12B—C12—H12C	109.5
N1—C1—H1	117.9		

### Hydrogen-bond geometry (Å, º)

**Table:**

*D*—H···*A*	*D*—H	H···*A*	*D*···*A*	*D*—H···*A*
O5—H1*O*5···O4^i^	0.83	1.92	2.734 (4)	165
O5—H2*O*5···O2^ii^	0.96	1.94	2.794 (4)	147
O6—H1*O*6···O1	0.94	1.90	2.820 (4)	167
O6—H2*O*6···O3^iii^	0.83	1.95	2.765 (4)	165
O7—H1*O*7···O4^ii^	0.83	1.89	2.722 (4)	175
O7—H2*O*7···O2	0.82	1.89	2.697 (4)	167
O8—H1*O*8···O1^iii^	0.77	1.95	2.719 (4)	175
O8—H2*O*8···O3^i^	0.89	1.91	2.792 (5)	174
C4—H4···O4^iv^	0.93	2.54	3.232 (5)	132

Symmetry codes: (i) *x*, *y*+1, *z*; (ii) −*x*+2, *y*+1/2, −*z*+3/2; (iii) −*x*+1, *y*+1/2, −*z*+3/2; (iv) *x*, −*y*−1/2, *z*+1/2.
